# Utility of the HScore for Predicting COVID-19 Severity

**DOI:** 10.7759/cureus.31969

**Published:** 2022-11-28

**Authors:** William Hannah, Anthony Shadiack, Melissa Markofski, Kevin Dao, Eric Shaw, Craig Odum, Nayda Parisio-Poldiak, Alexis Finer, Mike Flynn

**Affiliations:** 1 Graduate Medical Education, Memorial Health University Medical Center, Savannah, USA; 2 Family Medicine, Grand Strand Medical Center, Myrtle Beach, USA; 3 Health and Human Performance, University of Houston, Houston, USA; 4 Internal Medicine, Grand Strand Medical Center, Myrtle Beach, USA; 5 Graduate Medical Education, Coliseum Medical Centers, Macon, USA; 6 Graduate Medical Education, Grand Strand Medical Center, Hospital Corporation of America (HCA) Healthcare, Myrtle Beach, USA; 7 Data Analytics, Optum, Eden Prairie, USA; 8 Graduate Medical Education, Hospital Corporation of America (HCA) Healthcare, Charleston, USA

**Keywords:** covid-19 retro, anti-cytokine, cytokine storm, covid-19, hscore

## Abstract

Background: Cytokine release syndrome is a life-threatening condition known to cause fever and multiple organ dysfunction and is suspected to be related to the severity of coronavirus disease 2019 (COVID-19). We sought to examine the utility of the HScore and non-cytokine markers of inflammation for predicting COVID-19 outcomes. We hypothesized that cytokine storm, assessed by a modified HScore, would be linked to more severe COVID-19 symptoms and higher mortality.

Methods: A retrospective review of records from a large, private hospital system was conducted on patients with hemophagocytic lymphohistiocytosis (HLH) (2014-2019) and compared to a large cohort of COVID-19-positive patients (2020). Patients with a sufficient number of elements in their record for a modified HScore calculation (n=4663), were further subdivided into population 1 (POP1, n=67; HLH, n=493 COVID-19), which had eight HScore elements, and population 2 (POP2) with six available HScore elements (POP2, n=102; HLH, n=4561 COVID-19).

Results: Modified HScore predicted COVID-19 severity in POP1 and POP2 as measured by higher odds of being on a ventilator (POP2 OR: 1.46, CI: 1.42-1.5), ICU admission (POP2 OR: 1.38, CI: 1.34-1.42), a longer length of stay (p<0.0001), and higher mortality (POP2 OR: 1.34, CI: 1.31-1.39). C-reactive protein (CRP) and white blood cell (WBC) count were the most consistent non-cytokine predictors of COVID-19 severity.

Conclusion: Cytokine storm, evaluated using a modified HScore, appeared to play a role in the severity of COVID-19 infection, and selected non-cytokine markers of inflammation were predictive of disease severity.

## Introduction

Early in the coronavirus disease 2019 (COVID-19) pandemic, Mehta et al. proposed that the severity of symptoms and mortality rate for COVID-19 was linked to what is referred to as a cytokine storm [1]. The inflammatory storm is initiated by activated alveolar macrophages, initially producing large amounts of cytokines, triggering hyper-activation of immune cells [2,3]. Cytokine storm appears to be a significant contributor to the pathogenesis of severe cases of COVID-19 [3], and neutralizing inflammatory agents have been suggested as a means to reduce mortality [2].

Traditional markers of inflammation are universally elevated in cytokine storm and may also prove helpful in predicting the onset of hyper-inflammation in COVID-19 that can lead to multiorgan dysfunction [1]. Cui et al. found that a ≥50% reduction in CRP within three days of initiating corticosteroids strongly predicted reduced mortality in patients with COVID-19 [4]. Carubbi et al. found after following 61 COVID-19 patients that ferritin levels above the 25th percentile were helpful in determining lung involvement but were not associated with COVID-19 outcomes [5]. There are other reports of ferritin’s usefulness in predicting survival of COVID-19 patients [6,7].

Depending on the underlying etiology of cytokine storm, different grading systems have been used to predict and assess severity [3,8]. For example, certain biomarkers can be used to assess the severity of cytokine storm induced by chimeric antigen receptor (CAR) T-cell therapy [3]. Reactive hemophagocytic syndrome or secondary hemophagocytic lymphohistiocytosis (sHLH) is an example of cytokine storm, which can be related to infection, autoimmune disease, malignancy, and others, particularly in the setting of underlying immunosuppression. Fardet et al. developed the HScore, which can be used to estimate an individual’s risk of having HLH [8]. The cytokine storm in HLH is characterized by the proliferation and activation of lymphocytes and histiocytes, hyper-inflammation, cytopenias, and elevated inflammatory cytokines but can be significantly influenced by the specific driver of the syndrome [8].

The purpose of our study was to further examine the utility of a modified HScore and non-cytokine markers of inflammation for predicting COVID-19 outcomes. We also sought to assess the influence of anti-cytokine medications. We hypothesized that cytokine storm, as assessed by the modified HScore, would lead to a more severe progression of COVID-19 symptoms and be linked to higher mortality. We also hypothesized that non-cytokine markers of inflammation would predict the course of the disease. Further, we proposed that patients on anti-cytokine medications would be less susceptible to cytokine storm and have decreased disease severity.

## Materials and methods

We conducted a retrospective study of adults (≥18 years) admitted to the hospital (n=93,380) who were either COVID-19 positive (January-August 2020) or diagnosed with HLH (2014-2019). HLH-2004 is typically the criteria used to diagnose HLH; however, it is only approved for children and some of the laboratory markers needed are difficult to obtain [9,10]. Therefore, the HScore was used to confirm the hyper-inflammatory states that can also be observed in HLH [11,12].

**Figure 1:**
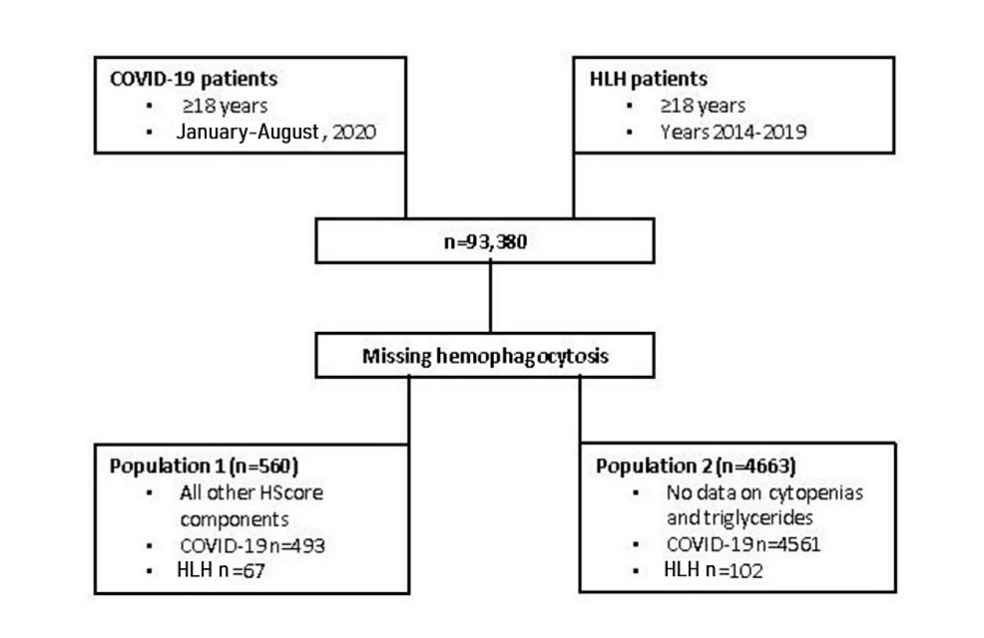
Flow diagram of group assignment. Data were collected from the HCA Healthcare database and grouped based on diagnosis (COVID-19 or HLH) and availability of HScore components. HLH: hemophagocytic lymphohistiocytosis; HCA: Hospital Corporation of America; COVID-19: coronavirus disease 2019

For the purposes of creating a modified HScore, COVID-19-positive patients (N=93,380) were grouped according to the available HScore elements. Hemophagocytosis on bone marrow is not typically measured nor ethical to obtain on COVID-19 patients. Thus, we identified 493 COVID-19 patients and 67 HLH patients with all HScore elements measured except hemophagocytosis on bone marrow (population 1 {POP1}) (Figure 1). Population 2 (POP2) patients had all HScore elements measured except hemophagocytosis on bone marrow, cytopenias, or triglycerides (COVID-19, n=4561; HLH, n=102).

Home and Inpatient Medications

The predominant drugs in our inpatient (prescribed) and home medication analyses were dexamethasone and prednisone. We also included a third category for “other anti-cytokine.” We searched for a broad list of biologics, steroids, and known immunosuppressives; however, few patients were taking biologic medications (e.g., tocilizumab).

Data Collection

The dataset was created from 186 hospitals in HCA Healthcare from 21 states across the United States, specifically by using Teradata SQL and the HCA database (IRB exempt determination, approval #2020-230). COVID-19-positive patients (polymerase chain reaction {PCR} test) and HLH patients (International Classification of Diseases {ICD}-10 codes) with multiple HScore elements available were included in the data set. A modified HScore was calculated using - known underlying immunosuppression (yes, no), temperature, organomegaly, the number of cytopenias, ferritin, triglyceride, fibrinogen, and aspartate aminotransferase (AST). All laboratory values were obtained from the samples first collected after patients were admitted to the hospital.

Data Analyses

The primary outcomes of this study were ventilator (required), ICU admission, length of stay (LOS), and in-hospital mortality. We sought to examine the effectiveness of a modified HScore for predicting these endpoints in COVID-19 patients. The secondary objective was to determine the influence of anti-cytokine medication exposure on these outcomes and the predictive ability of non-cytokine markers of inflammation. T-tests were used to determine significant differences in mean modified HScore between the HLH and COVID-19-positive populations. All regression analyses were completed for the COVID-19-positive population. COVID-19-positive patients were grouped into two populations based on the availability of HScore measures.

Two overarching analyses were conducted. First, logistic regression was used to determine the relationships between the primary outcomes (ventilator required, ICU admission, and mortality) and modified HScore in POP1 and POP2. Linear regression was used to predict LOS by modified HScore. Logistic regression was also employed to determine the influence of anti-cytokine medication exposure on the primary outcomes, with linear regression used to predict modified HScore and LOS by anti-cytokine medication exposure.

Second, logistic regression was used to predict the influence of non-cytokine markers of inflammation (ferritin, erythrocyte sedimentation rate {ESR}, c-reactive protein {CRP}, WBC, and platelets) on the primary outcomes - ventilator, ICU admission, and mortality. Initial lab values were used for non-cytokine markers of inflammation. Linear regression was used to predict LOS by non-cytokine markers of inflammation. Labs were interpreted on a 1-unit scale except for ferritin, which was interpreted on a 100-unit scale.

In our regression model for anti-cytokine medications (POP2 only), we included the anti-cytokine medications of COVID-19 patients (n=4031) which were given by inpatient pharmacy (INP), the medications patients with COVID-19 were taking on admission (HOME), or any exposure to anti-cytokine medications, either inpatient or home (ANY). We searched for a broad range of medications, but the most frequent medications were dexamethasone and prednisone. Thus, we included dexamethasone, prednisone, and “other” anti-cytokine drugs as covariates. The “other” anti-cytokine medications for INP were methylprednisolone (n=1295), hydrocortisone (n=231), and tocilizumab (n=10). We did not include any patients taking more than one of these medications in these analyses. In HOME meds (n=69), the other anti-cytokines were biologics (n=10, adalimumab or etanercept), methylprednisolone, hydrocortisone, and methotrexate.

All models controlled for the demographic variables age, sex, and race and were performed in both COVID-19-positive POP1 and POP2, with the exception of the multinomial models, which were only run in POP2 due to the small size of population POP1. All analyses were performed using SAS statistical software, version 9.4 (SAS Institute Inc., Cary, NC). Categorical variables were summarized using frequencies and percentages, while descriptive statistics for continuous variables included mean, median, standard deviation, and minimum and maximum values. An alpha level of 0.05 was used for type I error rate.

## Results

COVID-19 patients were older than HLH patients for both POP1 and POP2, but there were similar proportions of males and females across COVID-19 and HLH (Table 1). In the unadjusted analysis, length of stay (LOS) was similar between HLH and COVID-19 patients for POP1, but LOS was shorter for our POP2 COVID-19 patients (Table 1). Mechanical ventilation requirements for POP2 were not different between COVID-19 and HLH patients (Table 1). Mortality rates were higher for HLH patients in POP 1, but no significant differences in mortality were evident for the POP2 group (Table 1). Modified HScores were significantly (p<0.0001) higher for HLH patients than COVID-19 patients for POP1 (HLH, 190.5±46.8; COVID-19, 132.8±40.1) and POP2 (HLH, 97.6±47.9; COVID-19, 38.0±26.8).

**Table 1:** Descriptive statistics for the populations created by available HScore elements. POP1 includes HLH and COVID-19-positive patients with all HScore elements measured except HLH. POP2 included HLH and COVID-19-positive patients with all HScore elements measured except hemophagocytosis, cytopenias, and triglycerides. LOS: length of stay; ICU: intensive care unit; COVID-19: coronavirus disease 2019; PO1: population 1; PO2: population 2; HLH: hemophagocytic lymphohistiocytosis

Characteristic		POP1: COVID-19 (n=493)	POP1: HLH (n=67)	p-Value	POP2: COVID-19 (n=4561)	POP2: HLH (n=102)	p-Value
Sex, n (%)	Male	299 (60.7)	44 (65.7)	0.43	2551 (55.9)	63 (61.8)	0.24
	Female	194 (39.4)	23 (34.3)		2010 (44.1)	39 (38.2)	
Race/ethnicity, n (%)	White	281 (57.0)	41 (61.9)	0.75	2323 (50.93)	64 (62.8)	0.06
	Black	90 (18.3)	10 (14.9)		1014 (22.2)	16 (15.7)	
	Other	122 (24.8)	16 (23.9)		1224 (26.8)	22 (21.6)	
Age mean±SD		63.6±13.05	51.8±16.1	<0.0001	61.8±16.2	50.6±18.1	<0.0001
LOS mean±SD		26.4±15.9	28.7±24.9	0.304	14.1±13.0	23.5±23.0	<0.0001
Ventilator	Yes, n (%)	402 (81.5%)	27 (40.3)	<0.0001	1503 (32.9)	31 (30.4)	0.586
	No, n (%)	91 (18.5%)	40 (59.7)		3058 (67.1)	71 (69.6)	
ICU admission	Yes, n (%)	42 (8.5)	25 (37.3)	<0.0001	2659 (58.3)	57 (55.9)	0.625
	No, n (%)	451 (91.5)	42 (62.7)		1902 (41.7)	45 (44.1)	
Mortality	Alive, n (%)	208 (42.2)	45 (61.2)	0.0001	3536 (77.5)	80 (78.4)	0.828
	Dead, n (%)	285 (57.8)	22 (32.8)		1025 (22.5)	22 (21.6)	
Modified HScore		132.8±40.1	190.5±46.8	<0.0001	38.0±26.8	97.6±47.9	<0.0001

Modified HScore as a Predictor of COVID-19 Outcomes

We found that the odds of being on a ventilator were predicted by the modified HScore in both POP1 and POP2 (OR: 1.46, CI: 1.42-1.50) (Table 2). Likewise, ICU admission and mortality were significantly related to the modified HScore in both POP1 and POP2 (Table 2). The modified HScore was also significantly related to LOS in POP1 and POP2, such that in POP2 for each 10-point increase in the modified HScore, the patients’ LOS would increase by 1.72 days (p<0.0001, Table 2).

**Table 2:** Odds ratio and confidence intervals (CI, logistic regression) or parameter estimate and p-value (linear regression) for demographic variables and HScore predicting outcomes in populations 1 and 2. POP1 includes HLH and COVID-19-positive patients with all HScore elements measured except hemophagocytosis. POP2 included HLH and COVID-19-positive patients with all HScore elements measured except hemophagocytosis, cytopenias, and triglycerides. *P-value is <0.0001. **P-value is <0.001. ***P-value is <0.01. ****P-value is <0.05. LOS: length of stay; ICU: intensive care unit; COVID-19: coronavirus disease 2019; PO1: population 1; PO2: population 2; HLH: hemophagocytic lymphohistiocytosis

	Ventilation (OR, 95% CI)	ICU admission (OR, 95% CI)	Mortality (OR, 95% CI)	LOS (estimate)
POP 1 (n=493)				
Age	1.01 (0.99-1.03)	1.01 (0.98-1.04)	1.02 (1.01-1.04)***	-0.15***
Sex (ref=male)	1.15 (0.71-1.85)	0.90 (0.46-1.76)	1.05 (0.72-1.52)	3.49****
Race (ref=White)				
Black	1.03 (0.55-1.94)	1.14 (0.49-2.66)	1.12 (0.68-1.83)	-2.49
Other	0.90 (0.51-1.59	1.64 (0.68-3.94)	0.96 (0.62-1.50)	2.52
HScore	1.18 (1.11-1.26)*	1.18(1.08-1.28)**	1.08 (1.03-1.14)***	0.80*
POP 2 (n=4561)				
Age	1.02 (1.011-1.020)*	1.009 (1.005-1.013)*	1.034 (1.028-1.039)*	0.05*
Sex (ref=male)	1.11 (0.96-1.27)	1.08 (0.95-1.22)	1.05 (0.90-1.23)	0.28
Race (ref=White)				
Black	0.838 (0.70-1.00)	0.72 (0.61-0.84)**	0.89 (0.73-1.08)	-1.81*
Other	0.89 (0.75-1.06)	0.89 (0.76-1.04)	1.06 (0.87-1.26)	-0.13
HScore	1.46 (1.42-1.50)*	1.38 (1.34-1.42)*	1.34 (1.30-1.38)*	1.72*

Influence of Anti-cytokine Medications on COVID-19 Outcomes

For POP2, INP dexamethasone use was associated with a 6.34 (p<0.0001) increase in the modified HScore, and exposure to other INP anti-cytokine meds was associated with a 13.96 (p<0.02) increase in the modified HScore (Table 3). Conversely, exposure to HOME dexamethasone (-13.91, p<0.0001) yielded a lower modified HScore (Table 3). Similar to INP, when any exposure was considered, other anti-cytokine was associated with a significantly higher modified HScore (9.85, p=0.013) (Table 3).

**Table 3:** Odds ratio and CIs (logistic regression) or parameter estimate and p-value (linear regression) for anti-cytokine exposure predicting outcomes for population 2. POP2 included HLH and COVID-19-positive patients with all HScore elements measured except hemophagocytosis, cytopenias, and triglycerides. *Significant parameter estimates or odds ratios. POP2: population 2; HLH: hemophagocytic lymphohistiocytosis; COVID-19: coronavirus disease 2019

	HScore (estimate, p-Value)	Ventilation (OR, 95% CI)	ICU admission (OR, 95% CI)	Mortality (OR, 95% CI)	LOS (estimate, p-Value)
Population 2 (n=4031)					
Inpatient pharmacy (INP) model					
Dexamethasone	6.34* (<0.0001)	1.97* (1.61-2.41)	1.82* (1.46-2.25)	1.72 (1.38-2.14)	3.47* (<0.0001)
Prednisone	4.86 (0.0585)	1.43 (0.95-2.10)	1.81* (1.19-2.74)	0.70 (0.42-1.18)	10.35* (<0.0001)
Other anti-cytokine	13.96* (0.0256)	5.90* (2.09-16.64)	2.68 (0.88-8.18)	2.38 (0.92-6.19)	16.23* (<0.0001)
Home (HOME) medications model					
Dexamethasone	-13.92* (<0.0001)	0.33* (0.21-0.54)	1.09 (0.76-1.57)	0.43* (0.24-0.75)	-6.01* (<0.0001)
Prednisone	-2.58 (0.243)	0.92 (0.64-1.31)	1.07 (0.76-1.50)	1.10 (0.74-1.63)	-0.16 (0.88)
Other anti-cytokine	6.60 (0.20)	0.54 (0.22-1.35)	0.47 (0.22-1.02)	1.22 (0.53-2.82)	-1.29 (0.60)
Any (ANY) exposure model (inpatient pharmacy or home medications)					
Dexamethasone	1.68 (0.16)	1.42* (1.18-1.71)	1.65* (1.36-1.99)	1.36* (1.11-1.67)	1.39* (0.016)
Prednisone	0.85 (0.62)	1.15 (0.88-1.51)	1.39* (1.07-1.81)	0.95 (0.69-1.31)	4.63* (<0.0001)
Other anti-cytokine	9.85* (0.0134)	1.56 (0.86-2.84)	0.94 (0.52-1.71)	1.66 (0.88-3.11)	6.09* (0.0014)

INP dexamethasone vs. no anti-cytokine medication (reference value) was related to significantly higher odds of being on a ventilator, ICU admission, and a longer LOS (Table 3). There were also higher odds of ICU admission and longer LOS (10.35 days, p<0.0001) for INP prednisone (Table 3). There was a lower odds ratio for being on a ventilator (0.33, 0.20-0.54), mortality (0.43, 0.24-0.75), and shorter LOS (-6.01, p<0.0001) for HOME dexamethasone, but no similar, significant effects for HOME prednisone or HOME other anti-cytokine (Table 3).

The ANY exposure model (either home or inpatient) for dexamethasone, revealed significantly higher odds of being on a ventilator, ICU admission, mortality, and a longer LOS (Table 3). The ANY exposure model for prednisone revealed significantly higher odds of ICU admission, and a longer LOS (Table 3). Finally, ANY exposure model for other anti-cytokine was significantly related to the modified HScore and longer LOS (Table 3).

Non-cytokine Markers of Inflammation and COVID-19 Outcomes

For POP1, CRP significantly predicted ventilator requirement (1.04, 1.01-1.08), but was not significantly related to other outcome variables. CRP was a consistent predictor of COVID-19 outcomes in POP2, such that odds of requiring a ventilator (1.07, 1.06-1.08), ICU admission (1.08, 1.07-1.09), and mortality (1.05, 1.04-1.06) were significantly higher (Table 4). In addition, CRP predicted a shorter LOS in POP2, such that each unit increase in CRP was associated with a 0.31 (p<0.0001) shorter LOS (Table 4).

**Table 4:** Odds ratio and CIs (logistic regression) or parameter estimate and p-value (linear regression) for non-cytokine markers of inflammation predicting outcomes for POP1 and POP2. Here “n” denotes the number of subjects for which that lab was available. Ferritin was calculated as “per 100 unit increase.” *Significant parameter estimates or odds ratios denoted with. ESR: erythrocyte sedimentation rate; CRP: c-reactive protein; POP2: population 2; LOS: length of stay

	Ventilation (odds ratio)	ICU admission (odds ratio)	Mortality (odds ratio)	LOS (p-Value)
Population 1 (n=493)				
Ferritin (n=465)	1.01 (0.99-1.03)	1.00 (0.97-1.02)	0.98* (0.97-0.998)	0.048 (0.44)
ESR (n=195)	0.99 (0.98-1.01)	1.00 (0.98-1.02)	1.00 (0.99-1.01)	-0.015 (0.66)
CRP (n=414)	1.04* (1.01-1.08)	1.03 (0.98-1.08)	1.01 (0.99-1.047)	0.117 (0.20)
WBC (n=407)	1.04 (0.99-1.09)	1.09* (1.01-1.18)	1.02 (0.98-1.05)	0.059 (0.67)
Platelets (n=492)	1.004* (1.001-1.007)	1.003 (0.999-1.007)	1.002 (1.000-1.004)	0.023* (0.003)
Population 2 (n=4561)				
Ferritin (n=4275)	1.04* (1.030-1.044)	1.04* (1.032-1.050)	1.03* (1.022-1.036)	0.140* (<0.0001)
ESR (n=1441)	1.01* (1.002-1.009)	1.01* (1.003-1.010)	1.00 (0.999-1.006)	0.033* (0.00%)
CRP (n=3707)	1.07* (1.058-1.078)	1.08* (1.070-1.091)	1.05* (1.044-1.064)	0.310* (<0.0001)
WBC (n=3783)	1.07* (1.06-1.09)	1.09* (1.07-1.11)	1.04* (1.03-1.067)	0.293* (<0.0001)
Platelets (n=4520)	1.000 (0.999-1.001)	1.000 (0.999-1.001)	0.998* (0.998-0.999)	0.001 (0.70)

WBC was also a consistently significant predictor of COVID-19 outcomes in POP2, with higher odds of requiring a ventilator (1.07, 1.06-1.09), ICU admission (1.09, 1.07-1.11), and mortality (1.04, 1.03-1.06) (Table 4). Other non-cytokine markers revealed mixed results for relationships among ventilator, ICU admission, or mortality, with ESR for POP2 revealing relatively consistent relationships (Table 4).

## Discussion

We found that the modified HScore was a predictor of COVID-19 severity and that non-cytokine markers of inflammation were linked to the severity of COVID-19 symptoms. In prior studies, non-cytokine inflammatory markers such as CRP, ESR, ferritin, and WBC were universally elevated and related to the severity of cytokine storm [3,13]. In a meta-analysis of 56 studies and 8719 COVID-19 patients, Ji et al. demonstrated that patients with severe COVID-19 had significantly higher WBC, CRP, ESR, and procalcitonin (PCT) [14]. In another meta-analysis of 16 studies and 3962 COVID-19 patients, Zeng et al. showed that patients with less severe disease had lower levels of CRP, PCT, and ESR [15]. Finally, Cheng et al. performed a meta-analysis on a cumulative 10,614 COVID-19 patients and found significantly increased ferritin in patients with severe COVID-19 compared to less severe patients [6]. In Cheng et al. study, as in our analysis, hyper-ferritinemia was also associated with increased mortality.

After assessing non-cytokine markers of inflammation in COVID-19 patients (POP2), we found that for every 1-point increase in ESR, CRP, and WBC, there was statistical worsening in ventilation, ICU admission, and mortality (ESR not significant). In determining the severity of COVID-19 progression, initial non-cytokine markers, particularly ferritin, ESR, CRP, and WBC were the strongest predictors of disease severity. As an example, when controlling for age, sex, and race, for every 100-point increase in ferritin, a patient was 1.04 times more likely to be put on a ventilator, 1.04 times more likely to be admitted to the ICU, and 1.21 times more likely to die, compared to a patient with an initial ferritin value 100 points lower. We also found that LOS was 0.14 days longer in patients for every 100-point increase in ferritin (p<0.0001). Thus, our results are consistent with the published literature and provide further evidence of the relationship between selected non-cytokine inflammatory markers and COVID-19 severity [3,6].

In up to 20% of cases, severe COVID-19 infection is manifested by fever and pneumonia, leading to acute respiratory distress syndrome (ARDS) [16]. ARDS is thought to be mediated through an inflammatory cascade known as cytokine storm and is reminiscent of secondary or reactive hemophagocytic syndrome. In about 35% of adults, reactive hemophagocytic syndrome is due to viral infection, though it is unclear whether this immune dysregulation is from a failure to resolve the inflammatory process through ongoing viral replication or whether the immune hyper-reactivity simply underlies severe infection [3,17]. Yang et al. demonstrated that 17% of patients with COVID-19 have secondary HLH as diagnosed with an HScore greater than 169 [9]. Regardless, severe COVID-19 infection and reactive hemophagocytic syndrome share similar characteristics.

We found a significant relationship between a modified HScore and the severity of COVID-19 outcomes. The odds of being on a ventilator were also predicted by the modified HScore, and both ICU admission and mortality were significantly related to the modified HScore. The modified HScore was also significantly related to LOS, such that in POP2, for each 10-point increase in the modified HScore, the patient’s LOS would increase by 1.72 days (p<0.001). It is important to clarify that our modified HScore for POP1 did not include hemophagocytosis and therefore yielded lower than expected mean scores for both our COVID-19 patients (132.8±40.1) and HLH patients (190.5±46.8). POP2 also excluded triglycerides and the number of cytopenias from the HScore calculation, and as expected, we had lower mean scores for both COVID-19 (37.9±29.8) and HLH patients (97.6±47.9). Despite these missing elements, the modified HScore was predictive of COVID-19 severity.

Multiple authors have suggested using the HScore to assess for HLH in patients with COVID-19; however, there are concerns about utilizing this score due to the absence of leukopenia in COVID-19 infection, relatively lower serum ferritin as compared to HLH, and the inability to obtain pathologic evidence of hemophagocytosis [9,17-20]. Regardless, COVID-19 and HLH share core features that reflect a common immune response that can be utilized to assess severity. In a multicenter prospective study of 193 hospitalized children and adults, Bordbar et al. evaluated the HScore as a predictor of disease in COVID-19 patients [21]. The authors did not perform bone marrow aspiration due to ethical concerns and thus recorded a zero for missing data in the HScore calculation. Similar to our study, these authors noted a lower HScore in COVID-19 patients compared with HLH patients; however, a higher HScore in COVID-19 patients was associated with a higher rate of ICU admission, increased mortality, and longer length of stay. Our results are consistent with those in the Bordbar et al. study and suggest that the HScore (even when modified without a bone marrow biopsy demonstrating hemophagocytosis) can be utilized to identify COVID-19 patients with the most severe disease and potentially those who are more likely to benefit from immunosuppressive treatment [21].

Horby et al. established that dexamethasone reduced mortality among the most severe COVID-19 cases, characterized by elevated CRP and need for supplemental oxygen [22]. Further, a meta-analysis of seven randomized trials by Sterne et al. showed lower all-cause mortality at 28 days in COVID-19 patients treated with glucocorticoids compared to usual care [23]. Recently, two larger clinical trials assessed the efficacy of IL-6 receptor antagonists in patients with COVID-19 with mixed results [24,25]. Thus, it appears the effectiveness of corticosteroids in the treatment of severe COVID-19 is clear; however, there remains a need to define subgroups of patients with COVID-19 who may benefit from IL-6 receptor blockade.

Based on these data, we posited that patients on immunosuppressive treatment, either at home or in the inpatient setting, may decrease the risk of COVID-19 severity by preventing cytokine storm. Few patients in our study received anti-cytokine medications other than corticosteroids. Nevertheless, our results confirm that exposure to these medications as an inpatient was associated with a higher modified HScore and higher odds of requiring mechanical ventilation, being admitted to an ICU, longer LOS, and higher mortality. We interpret these results to suggest that patients who are more ill are more likely to be prescribed corticosteroids as an inpatient. However, for HOME meds, patients taking dexamethasone and prednisone had a lower modified HScore, lower odds of being on a ventilator, and shorter LOS. HOME dexamethasone was also associated with a lower odds of death. These relationships warrant replication as the overall number of patients taking qualifying home meds was low.

Limitations

Among the limitations of our study was our focus on HScore as a marker of COVID-19 severity, with several components of the HScore not reported. The HScore also relies on cytopenias, including leukopenia, which is often not observed in COVID-19 patients. As a result, we utilized a modified HScore to capture a larger cohort of patients, which resulted in lower overall scores. Another limitation was that non-cytokine inflammatory markers were assessed on admission and may not have adequately captured disease severity as it progressed throughout a lengthy hospitalization. Also, missing data contributed to a much smaller sample for POP1, which may have introduced bias. Finally, due to the low frequency of medications other than corticosteroids, we were unable to capture the effects of other anti-cytokine medications on patient outcomes.

## Conclusions

The HScore, used to determine the hyper-inflammatory response of HLH, may be a valuable tool in assessing COVID-19 severity. Consistent with prior studies, non-cytokine inflammatory markers were related to disease severity and overall mortality. Further research should be directed at identifying the potential benefits of anti-cytokine therapy other than corticosteroids, especially the appropriate time to initiate therapy and the subpopulations that may benefit most.
